# A rare discovery of Spigelian-cryptorchidism syndrome in adult

**DOI:** 10.1016/j.eucr.2021.101687

**Published:** 2021-04-20

**Authors:** Rajan Gurung, Aishath Azna Ali, Fei Yee Lee, Aung Mra, Firdaus Hayati

**Affiliations:** a Department of Surgery, Indira Gandhi Memorial Hospital, Male’, Maldives; b Department of Urology, Faculty of Medicine and Health Sciences, Universiti Putra Malaysia, Seri Kembangan, Selangor, Malaysia; c Department of Surgery, Faculty of Medicine and Health Sciences, Universiti Malaysia Sabah, Kota Kinabalu, Sabah, Malaysia

**Keywords:** Cryptorchidism, Hernia, Ventral hernia, SH, Spigelian hernia

## Abstract

Spigelian hernia (SH) occurs due to the protrusion through a congenital or acquired defect or weakness in the Spigelian aponeurosis. SH accounts for only 0.1–0.4% of occurrence and a 17–24% risk of strangulation. We hereby report a case of a 34-year-old gentleman presented with concomitant incarceration of the omentum with small intestine and testis in Spigelian hernia sac. We have successfully operated on this patient via a transperitoneal approach with a small incision over the hernia site. This incision could be an alternative to midline laparotomy as a safe and effective method in managing incarcerated SH in an emergency setting.

## Introduction

Spigelian-cryptorchidism syndrome is mostly reported in the paediatric age group and they are commonly presented with swelling of the abdominal wall or intestinal obstruction with associated ipsilateral cryptorchidism.^1^ Spigelian hernia (SH) accounts for only 0.1%–0.4% of occurrence and a 17%–24% risk of strangulation.^2^ SH occurs after a protrusion of preperitoneal fat, a sac of the peritoneum or an organ, through a congenital or acquired defect or weakness in the Spigelian aponeurosis. This aponeurosis is formed as part of the aponeurosis of the transverse abdominal muscle bounded by the linea semilunaris laterally and the lateral part of the rectus muscle medially.^1^ There are many associated risk factors, including increased intra-abdominal pressure (e.g., obesity, disorders of collagen, smoking, or chronic obstructive pulmonary disease), trauma or previous surgery. SH can contain various visceral organs, including small bowel, omentum, large bowel, stomach, gallbladder, Meckel diverticulum, ovary, leiomyoma of the uterus, bladder, and even the testis.^1^ The association with undescended testes is poorly understood. The late discovery of Spigelian-cryptorchidism syndrome in the patient in this research is rare, in which he was a commoner in paediatric age group. This work reports a case of a 34-year-old man presented with concomitant incarceration of the omentum with small intestine and testis in Spigelian hernia sac.

## Case report

A 34-year-old gentleman was presented to the emergency department with a one-day history of painful irreducible swelling over the right lumbar region, in which he was associated with several episodes of vomiting but without flatus. Upon further questioning, the swelling over the right lumbar region appeared intermittently upon straining since childhood. It was associated with mild discomfort and occasionally, the aforementioned patient would notice the pain radiates to the groin when there is swelling. He denies any history of constipation or abdominal distension previously. He was diagnosed with right undescended testes since birth; however, he was unsure of any intervention performed previously. He is, otherwise, not known to have any comorbidity. He works as a manual labourer, denies consuming any alcohol, but smokes one pack a year for the past 14 years. He was born term with no significant birth history.

**Fig. 1:**
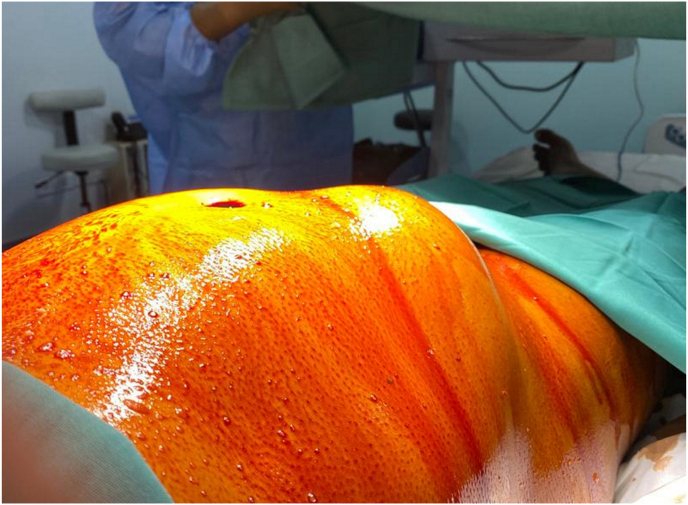
A swelling measuring a size of a fist was visualized over the right lumbar region despite muscle relaxation.

**Fig. 2:**
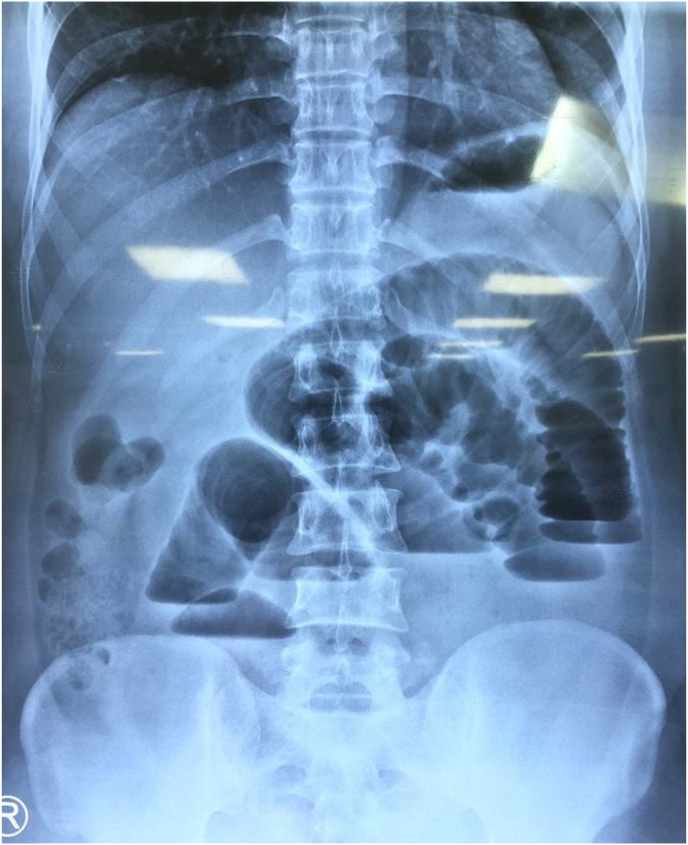
Abdominal radiography at erect position demonstrated multiple air fluid levels with prominent valvulae conniventes, which represents a small bowel obstruction.

Upon presentation in the emergency department, he was stable and not septic looking. Additionally, he was hemodynamically stable. A firm, tender, non-fluctuant, non-pulsatile, irreducible mass measuring 5 × 10 cm in size was identified (Fig. 1). There was no change in the overlying skin colour. The cough impulse was not demonstrable. The right testis was not palpable within the scrotal sac or along the inguinal region, whereas the left testis is normal and located in the scrotum. The laboratory investigations were unremarkable. However, the abdominal radiography demonstrated features suggestive of small bowel obstruction (Fig. 2).

**Fig. 3:**
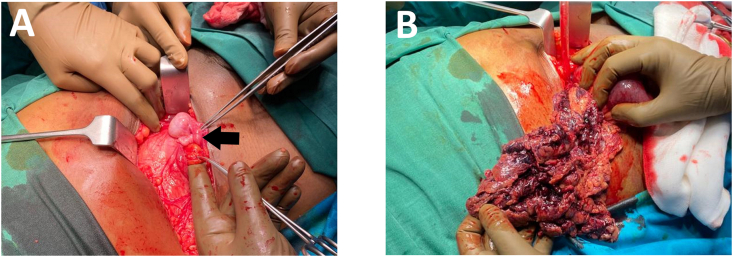
(A) Right cystic atrophic testis (arrow) was discovered in the hernial sac; and (B) Gangrenous omentum within the hernial sac resulted with omentectomy.

He underwent emergent open hernia repair with an oblique incision over the swelling. Intraoperatively, a defect on the lateral border of the rectus abdominis muscle was found along the arcuate line with the hernia sac measuring 6 × 10 cm containing 25 ml of serous fluid, gangrenous omentum, dusky terminal ileum with cystic atrophic testes (Fig. 3A and B). No gubernaculum was identified. The strangulated bowel was viable after resuscitation. The gangrenous omentum was resected along with the atrophied testes and hernioplasty performed. The patient recovered well postoperatively and was discharged on Day 5. The histopathology report was consistent with gangrenous omentum and atrophied testes.

## Discussion

The presentation of SH can be very vague and may vary. Most cases are asymptomatic, which may be detected for an unrelated reason, or they may present pain or a bulge in the lower abdomen in the region of Spigelian aponeurosis. If left untreated, the hernia may become incarcerated and strangulated. In a recent study, approximately 27% of patients with SH required emergency surgery for strangulation or incarceration.^3^

Clinical examination is the mainstay of the diagnosis. The vast majority of SH lie in the SH belt, a transverse zone of 6 cm above the interspinal plane, which is at the Spigelian belt. SH may sometimes be misdiagnosed as a direct inguinal hernia given its exact location. In some cases, the hernia may not be apparent, even with positioning or Valsalva manoeuvre; this is especially true in obese patients. Therefore, a physical exam alone may only accurately diagnose this rare hernia in one-half of patients.^4^ Hence, with the advent of imaging, abdominal ultrasound or computed tomography may be utilised as an adjunct in performing diagnosis of this rare type of hernia. Imaging also helps to delineate the anatomy and size of the hernia.^4^ However, in an emergency situation, imaging is not necessary. As for the patient in this work, he was put under general anaesthesia and the hernia was repaired on the same day without imaging. Exploration was done on the hernia via a transverse incision made over the hernia and adequate exposure and assessment were achieved.

The mainstay of management of this anomaly includes repair of the hernia and scrotal placement of the undescended testes. One should remember to look for undescended testes within the hernia sac in the case of SH, with no testes found within the scrotal sac or inguinal region. Embryologically, the gubernaculum is a prerequisite for the development of the inguinal canal. For the patient in this study, his testes were found to be atrophied and no gubernaculum was found; hence, it was assumed that there was a lack of an inguinal canal. The inguinal canal was not explored in the emergency setting. There are various techniques that have been reported in the management of the rare SH. Many have embarked on laparoscopic repair over the last three decades, with laparoscopic transabdominal preperitoneal repair with mesh reported to have the lowest recurrence and complication rate. The overall recurrence rate in laparoscopic repair was 2.8% and open repair was higher at 4.2%.^5^

## Conclusion

It can be concluded that the patient in this work is likely to have a congenital condition consistent with the findings of cryptorchidism with ipsilateral atrophied testes found in the SH sac. There are many ways to manage SH, depending on the expertise available in one centre although it was reported that open repair has a higher complication and recurrence rate. Managing an SH is almost equivocal to other types of hernia. Treating risk factors is of the utmost priority; however, in emergency circumstances, exploration is more trivial.
